# Significance of Low Muscle Mass on Arterial Stiffness as Measured by Cardio-Ankle Vascular Index

**DOI:** 10.3389/fcvm.2022.857871

**Published:** 2022-06-14

**Authors:** Hyo Eun Park, Goh Eun Chung, Heesun Lee, Min Joo Kim, Su-Yeon Choi, Wonjae Lee, Ji Won Yoon

**Affiliations:** ^1^Division of Cardiology, Department of Internal Medicine, Healthcare System Gangnam Center, Healthcare Research Institute, Seoul National University Hospital, Seoul, South Korea; ^2^Division of Gastroenterology, Department of Internal Medicine, Healthcare System Gangnam Center, Healthcare Research Institute, Seoul National University Hospital, Seoul, South Korea; ^3^Division of Endocrinology, Department of Internal Medicine, Healthcare System Gangnam Center, Healthcare Research Institute, Seoul National University Hospital, Seoul, South Korea; ^4^Division of Cardiology, Department of Internal Medicine, Seoul National University Bundang Hospital, Seongnam, South Korea

**Keywords:** sarcopenia, muscle mass, arterial stiffness, CAVI, atherosclerosis

## Abstract

**Aim:**

A link between low muscle mass and arterial stiffness is not always consistent. In this study, we aimed to evaluate the clinical significance of low skeletal muscle mass in relation to arterial stiffness measured by the cardio-ankle vascular index (CAVI).

**Methods:**

A total of 2,561 asymptomatic Korean subjects who underwent bioelectrical impedance analysis (BIA) and CAVI were included for analysis. Using appendicular skeletal muscle mass (ASM), classes I and II sarcopenia were defined as ASM% greater than 1 standard deviation (SD) and 2 SDs below the gender-specific mean of healthy young Korean adults.

**Results:**

Compared to normal, CAVI was significantly higher, but the number of patients with a low ankle-brachial index (ABI) was not significantly different (*p* < 0.001 for CAVI, *p* = 0.078 for ABI). Classes I and II sarcopenia showed an independent and significant association with CAVI (estimate 0.148, standard error (SE) 0.043, *p* < 0.001 and estimate 0.304, SE 0.073, *p* < 0.001 for classes I and II sarcopenia, respectively, adjusted for age groups, gender, body mass index (BMI) ≥25, hypertension, diabetes, hypercholesterolemia, and smoking).

**Conclusion:**

Low muscle mass is independently and significantly associated with increased CAVI, and should be considered when managing asymptomatic subjects to assess the risk of atherosclerosis.

## Highlights

- Arterial stiffness, as measured by the cardio-ankle vascular index (CAVI), is significantly higher in the presence of low muscle mass, even in apparently healthy populations.
- Low muscle mass is a parameter that is independently and inversely associated with CAVI, adjusting for age and known risk factors of atherosclerosis.
- Although an exact causal relationship is still to be elucidated, a decline in muscle mass should be of concern in the assessment of metabolic and cardiovascular risk even in apparently healthy populations, as CAVI is associated with various metabolic parameters, atherosclerosis, and cardiovascular diseases.

## Introduction

The decrease in skeletal muscle mass is one of the organ changes representative of the aging process. The progressive loss of the quantity and quality of skeletal muscle is related to various health problems in the aging society as sarcopenia is associated with metabolic parameters (1), physical dysfunction and dependence (2), and increased morbidity and mortality (3, 4). In addition to aging, cellular and molecular mechanisms, such as oxidative stress (5), inflammation, insulin resistance (6), and hormonal factors (7, 8), are known to play roles in the process of sarcopenia, which are also known as predisposing factors for atherosclerosis, resulting in increased cardiovascular morbidity and mortality. According to Campo et al. (9), the lack of muscles, rather than increased fatty tissue, is associated with subclinical atherosclerosis and endothelial dysfunction, suggesting that muscle mass and strength are the key determinants in patients with sarcopenia. Sarcopenia is becoming an unavoidable concern in Korea as well, as the number of elderly population is increasing at a rapid pace and the medical cost is rapidly becoming a significant social burden.

Arterial stiffness can be measured by various methods, such as pulse wave velocity (PWV) (10), pulse pressures (11), and cardio-ankle vascular index (CAVI) (12). The measurement of PWV is widely accepted, but study accuracy is affected by blood pressure during measurement. CAVI reflects the stiffness of the whole arterial segment, independent of blood pressure (13). Previous studies have shown a significant association of CAVI with coronary atherosclerosis (14), cardiac function (15), arrhythmias (16), cerebrovascular disease (17), and other metabolic parameters (18, 19). A meta-analysis of cross-sectional studies showed a negative correlation of muscle mass and PWV, with different ethnics and measurement methods (20), but the association between CAVI and muscle mass showed inconsistent results. In the Caucasian population, a strong association was found between CAVI and skeletal mass index (7), whereas in a Korean study, the significance of muscle mass deficit lost its power after adjusting for confounders (21). Preexisting ethnic difference in muscle mass, especially skeletal muscle mass (22), and the development or progression of atherosclerosis would affect the interpretation and significance of cardiovascular risk factors.

Thus, in this study, we aimed to evaluate the association between low muscle mass and arterial stiffness measured by CAVI, in asymptomatic Korean subjects.

## Methods

### Study Population

The study was based on retrospective review of medical records. Of all subjects who underwent a health check-up evaluation at Seoul National University Hospital Gangnam Center from 2018 to 2020, we retrieved 2,933 subjects with CAVI. Exclusion criteria were: other ethnicities (*n* = 173), no bioelectrical impedance analysis (BIA, *n* = 122), history of myocardial infarction (*n* = 23) or known angina (*n* = 40), and history of stroke (*n* = 14). A total of 2,561 asymptomatic Korean subjects without known cardiovascular or cerebrovascular disease were included in the analysis.

Comorbidities of each subject were reviewed based on subject-recorded questionnaires and medications. Hypertension was defined as measured blood pressure ≥ 140/90 mmHg or taking antihypertensive medications (23), diabetes was defined as taking any glucose-lowering agents, fasting glucose ≥126 mg/dl, or glycated hemoglobin (HbA1C) ≥ 6.5% (24), and hypercholesterolemia was defined as taking lipid-lowering agents or low-density lipoprotein (LDL) cholesterol ≥ 160 mg/dl (25, 26). Based on subject-recorded questionnaires, subjects were categorized as never smokers, ex-smokers, and current smokers. Ex-smokers were defined as those who had smoked more than 100 cigarettes in their lifetime but had not smoked in the last 28 days. Never smokers were those who had not smoked more than 100 cigarettes in their lifetime and did not currently smoke.

The study protocol conformed to the ethical guidelines of the 1975 Declaration of Helsinki and was approved by the Institutional Review Board of Seoul National University Hospital (IRB No. H-1606-102-771). Because the current study was performed with a retrospective design using a database and medical records, informed consent was waived by the Institutional Review Board of Seoul National University Hospital.

### Measurement of Anthropometric and Laboratory Parameters

Body weight, height, and waist circumference (WC) were measured on the day of the examination. Height and body weight were measured with a digital scale. WC was measured at the midpoint between the lower costal margin and the iliac crest by a well-trained nurse. Body mass index (BMI) was calculated using height and weight according to the formula: BMI = weight (kg)/height (m^2^).

All subjects were fasting for at least 12 h prior to laboratory tests. Blood tests included total cholesterol, triglyceride (TG), high-density lipoprotein (HDL) cholesterol, LDL cholesterol, fasting glucose, HbA1c, high-sensitivity C-reactive protein (hsCRP), blood urea nitrogen, and creatinine. Glomerular filtration rate (GFR) was calculated according to the Modification of Diet in Renal Disease (MDRD) equation as follows: GFR (ml/min/1.73 m^2^) = 186 × serum creatinine^−1.154^ × age^−0.203^ × 0.742 (if female).

### Body Composition Analysis

Bioelectrical impedance analysis with the InBody720 body composition analyzer (InBody Co., Ltd., Seoul, Korea) was used for body composition analysis. Subjects were in the standing position for 5–10 min with legs slightly separated and the arms slightly abducted from the trunk. Subjects were instructed to hold the handles of the analyzer, for contact with the electrodes on each limb. After stabilization of the measurements, impedance was provided for each segment, including the trunk and the four limbs, by performing multi-frequency measurements to estimate appendicular skeletal muscle mass (ASM) (27, 28).

### Definition of Sarcopenia

Appendicular skeletal muscle mass (kg) was calculated as the sum of lean muscle mass in the bilateral upper and lower limbs. ASM % was calculated as ASM/weight (kg) × 100, modified from Janssen's study (29). Class I sarcopenia was defined as ASM% beyond 1 standard deviation (SD) and <2 SD, and class II sarcopenia was defined as 2 SD below the gender-specific mean of healthy young adults according to the nationwide health examination of the Korean population (30). The cutoff values for ASM% were 32.2 in men and 25.6 in women for class I sarcopenia, and 29.1 in men and 23.0 in women for class II sarcopenia (30).

### Measurement of Arterial Stiffness Using CAVI

For the measurement of CAVI, A VaSera VS-1000 (Fukuda Denshi Co. Ltd., Tokyo, Japan) was used by the methods described previously (12, 14, 31). A 5-min rest was given before measurement in a sitting position. Brachial pulse pressure was measured with an automatic cuff oscillometric device, and the average of two readings was used to determine systolic and diastolic pressures and pulse pressure. In the supine position, cuffs were applied to four extremities at both upper arms and ankles and, after 10 min of rest, the measurement was performed (12, 14, 31). A phonocardiogram was placed at the border of the right sternum, in the second intercostal space, and electrocardiogram leads were attached to both wrists. Dividing the vascular length (*L*) by the time (*T*) taken for the pulse wave to propagate from the aortic valve to the ankle, PWV was calculated. *T* is calculated by summing the time between the rise of the brachial pulse wave and the rise of the ankle pulse wave and the time between the aortic valve closure sound and the notch of the brachial pulse wave. Using the following equation, CAVI was determined;


$$\text{CAVI}=\text{a}[(2\rho /\Delta \text{P})\times \times \text{ln}(\text{Ps}/\text{Pd})\times {\text{PWV}}^{2}]+\text{b}$$

where *Ps* and *Pd* are systolic and diastolic blood pressures, respectively, Δ*P* is *Ps-Pd*, ρ is the blood density, and a and b are constants (12). The ankle-brachial index (ABI) was measured during CAVI, and the mean value of the right and left CAVI was used for analysis.

### Statistical Analysis

Data are expressed as mean ± SD. Comparison of baseline characteristics according to normal, classes I and II sarcopenia was performed by one-way analysis of variance (ANOVA) for continuous variables. Independent *t*-test was performed for comparison of continuous variables between two groups. Categorical variables were compared by chi-squared analysis. Linear regression analysis was applied to evaluate the correlation of CAVI with each parameter, and a general linear model was used to evaluate the significance of classes I and II sarcopenia in determining CAVI, adjusting for age, and other known cardiovascular risk factors. For multivariate analysis, age was categorized into groups of 20 years, and the stepwise backward method was applied. For all statistical analyses, the statistical software package (SPSS 17.0, SPSS, Inc., Chicago, IL, USA) and *R* were used and a *p* < 0.05 was considered statistically significant.

## Results

The baseline characteristics of the study subjects are shown in Table 1. Class I and II sarcopenia groups were significantly older (*p* < 0.001) and obese (*p* < 0.001), and had a greater number of men (*p* < 0.001) compared to the normal group. Hypertension, diabetes, and hypercholesterolemia were more prevalent in both class I and II sarcopenia groups (*p* < 0.001 for all three comorbidities). Systolic and diastolic blood pressures and pulse pressure were higher in sarcopenia groups than in the normal group. Total cholesterol (*p* = 0.001), TG (*p* < 0.001), and HDL cholesterol (*p* < 0.001) showed significant differences among the three groups, whereas LDL cholesterol levels did not differ (*p* = 0.237). Both fasting glucose and HbA1C were higher in class I and II sarcopenia groups than in the normal group (*p* < 0.001 for glucose and HbA1C). An ABI <0.9 was found in 17 (1.2%) subjects in the normal group and 4 (0.5%) subjects with class I sarcopenia. None of the subjects with the class II sarcopenia group had a low ABI.

**Table 1:** Baseline characteristics of the study subjects.

**Parameters**	**Normal** **(*n* = 1,478)**	**Class I Sarcopenia** **(*n* = 875)**	**Class II Sarcopenia (*n* = 206)**	***p*** **-value**
Age as continuous variable	56 ± 9	58 ± 10^*^	60 ± 12^*^	<0.001
Male gender	943 (63.8%)	718 (82.1%)^*^	173 (84.0%)^*^	<0.001
Comorbidities				
Hypertension	299 (20.2%)	327 (37.4%)^*^	104 (50.5%)^*^^†^	<0.001
Diabetes mellitus	127 (8.6%)	124 (14.2%)^*^	47 (22.8%)^*^^†^	<0.001
Hypercholesterolemia	362 (24.5%)	323 (36.9%)^*^	79 (38.3%)^*^^†^	<0.001
Smoking				0.123
Never	1118 (75.6%)	616 (70.4%)	151 (73.3%)	
Ex smoker	86 (5.8%)	59 (6.7%)^*^	13 (6.3%)	
Current smoker	273 (18.5%)	198 (22.6%)^*^	41 (19.9%)	
Anthropometric parameters				
BMI, kg/m^2^	22.8 ± 2.3	25.6 ± 2.4^*^	27.9 ± 3.4^*^^†^	<0.001
BMI≥25	258 (17.5%)	521 (59.5%)^*^	173 (84.0%)^*^^†^	<0.001
Waist circumference, cm	84 ± 7	92 ± 7^*^	98 ± 8^*^^†^	<0.001
SBP, mmHg	119 ± 16	124 ± 15^*^	126 ± 17^*^	<0.001
DBP, mmHg	80 ± 11	82 ± 10^*^	83 ± 11^*^	<0.001
PP, mmHg	39 ± 12	42 ± 13^*^	43 ± 13^*^	<0.001
Laboratory parameters				
Total cholesterol, mg/dL	191 ± 36	185 ± 38^*^	188 ± 42^*^	0.001
Triglyceride, mg/dL	110 ± 67	137 ± 79^*^	150 ± 92^*^^†^	<0.001
HDL-cholesterol, mg/dL	56 ± 14	51 ± 12^*^	52 ± 16^*^	<0.001
LDL-cholesterol, mg/dL	123 ± 32	120 ± 34	121 ± 36	0.237
Glucose, mg/dL	103 ± 19	108 ± 21^*^	115 ± 29^*^^†^	<0.001
HbA1c, %	5.7 ± 0.6	5.9 ± 0.7^*^	6.1 ± 0.9^*^^†^	<0.001
Blood urea nitrogen, mg/dL	13.9 ± 3.7	14.5 ± 3.7^*^	14.6 ± 3.7^*^	<0.001
Creatinine, mg/dL	0.86 ± 0.19	0.90 ± 0.18^*^	0.86 ± 0.23	<0.001
GFR, mL/min/1.73m^2^	86 ± 16	86 ± 28	85 ± 21	0.750
hsCRP, mg/dL	0.10 ± 0.43	0.13 ± 0.27	0.16 ± 0.30	0.052
ASM%	32.0 ± 3.2	29.7 ± 2.6^*^	26.7 ± 3.0^*^^†^	<0.001
CAVI	7.8 ± 1.0	8.1 ± 1.1^*^	8.3 ± 1.4^*^	<0.001
ABI<0.9	17 (1.2%)	4 (0.5%)	0 (0%)	0.078

*ABI, ankle-brachial index; ASM, appendicular skeletal muscle mass; BMI, body mass index; CAVI, cardio-ankle vascular index; DBP, diastolic blood pressure; GFR, glomerular filtration rate; HbA1C, glycated hemoglobin; HDL, high density lipoprotein; hsCRP, high sensitivity C reactive protein; LDL, low density lipoprotein; PP, pulse pressure; SBP, systolic blood pressure*.

* *p < 0.05, compared to normal*.

† *p < 0.05, compared to class I sarcopenia*.

When class I and II sarcopenia were compared, the prevalence of hypertension and diabetes was significantly more common in class II sarcopenia (50.5 vs. 37.4%, *p* < 0.001 for hypertension, 22.8 vs. 14.2%, *p* < 0.001 for diabetes). BMI, WC, TG, glucose, and HbA1C levels were also significantly higher in class II sarcopenia than in class I sarcopenia (27.9 ± 3.4 vs. 25.6 ± 2.4, *p* < 0.001 for BMI; 98 ± 8 vs. 92 ± 7, *p* < 0.001 for WC; 150 ± 92 vs. 137 ± 79, *p* < 0.001 for TG; 115 ± 29 vs. 108 ± 21, *p* < 0.001 for glucose; and 6.1 ± 0.9 vs. 5.9 ± 0.7, *p* < 0.001 for HbA1C). CAVI did not differ significantly between class I and II sarcopenia (*p* = 0.066).

### Association of Each Parameter With Increasing Arterial Stiffness

Age, male gender, and the presence of hypertension, diabetes, and hypercholesterolemia were associated with increased arterial stiffness (Table 2). Systolic blood pressure and pulse pressure showed a positive correlation with CAVI (*p* < 0.001 for both). From blood tests, total cholesterol, LDL cholesterol, and GFR showed a negative correlation with CAVI, whereas glucose and HbA1C showed a positive association with CAVI. Decreased ASM% was significantly related to increased CAVI. CAVI was significantly higher in both class I and II sarcopenia groups (*p* < 0.001 for both class I and II sarcopenia) than in the normal group.

**Table 2:** The association of parameters in association with arterial stiffness.

	**Estimate**	**Standard Error**	***P*** **-value**
Age as continuous variable	0.069	0.002	<0.001
Age groups compared to 20–39			
Age 40–59	0.771	0.102	<0.001
Age ≥60	1.850	0.104	<0.001
Male gender	0.112	0.048	0.018
Comorbidities			
Hypertension	0.457	0.042	<0.001
Diabetes mellitus	0.754	0.054	<0.001
Hypercholesterolemia	0.359	0.043	<0.001
Smoking			
Ex smoker	−0.007	0.090	0.937
Current smoker	−0.155	0.054	0.004
Anthropometric parameters			
BMI≥25	−0.177	0.044	<0.001
Waist circumference, cm	0.004	0.003	0.087
SBP, mmHg	0.019	0.001	<0.001
DBP, mmHg	0.004	0.002	0.080
PP, mmHg	0.028	0.002	<0.001
Laboratory parameters			
Total cholesterol, mg/dL	−0.003	0.001	<0.001
Triglyceride, mg/dL	0.000	0.000	0.948
HDL cholesterol, mg/dL	−0.002	0.002	0.166
LDL cholesterol, mg/dL	−0.004	0.001	<0.001
Glucose, mg/dL	0.013	0.001	<0.001
HbA1c, %	0.422	0.030	<0.001
Blood urea nitrogen, mg/dL	0.060	0.006	<0.001
Creatinine, mg/dL	0.325	0.110	0.003
GFR, mL/min/1.73m^2^	−0.006	0.001	<0.001
hsCRP, mg/dL	0.077	0.058	0.184
ASM%	−0.024	0.006	<0.001
Sarcopenia compared to normal muscle group			
Class I sarcopenia	0.238	0.046	<0.001
Class II sarcopenia	0.433	0.080	<0.001

*ASM, appendicular skeletal muscle mass; BMI, body mass index; DBP, diastolic blood pressure; GFR, glomerular filtration rate; HbA1C, glycated hemoglobin; HDL, high density lipoprotein; hsCRP, high sensitivity C-reactive protein; LDL, low density lipoprotein; PP, pulse pressure; SBP, systolic blood pressure*.

### Significance of Sarcopenia in Relation to Arterial Stiffness

Multiple multivariate models were evaluated to adjust for known cardiovascular risk factors affecting CAVI. Model I, with age in groups, gender, and BMI ≥ 25 adjusted, showed that both class I and II sarcopenia were significant factors affecting CAVI (estimate 0.198, standard error (SE) 0.044, *p* < 0.001 for class I sarcopenia and estimate 0.424, SE 0.075, *p* < 0.001 for class II sarcopenia, Table 3). Further decrease in ASM, given by comparing class II to class I sarcopenia, still showed a positive association with arterial stiffness (estimate 0.241, SE 0.080, *p* = 0.003).

**Table 3:** Multivariate analysis to determine the significance of sarcopenia in arterial stiffness.

		**Estimate**	**Standard Error**	***P*** **-value**
Model I	Class I sarcopenia	0.198	0.044	<0.001
	Class II sarcopenia	0.424	0.075	<0.001
Model II	Class I sarcopenia	0.146	0.042	0.001
	Class II sarcopenia	0.302	0.073	<0.001
Model III	Class I sarcopenia	0.148	0.043	0.001
	Class II sarcopenia	0.304	0.073	<0.001

*Adjusted variables for model I; age, sex, and BMI ≥ 25*.

*Adjusted variables for model II: age, sex, BMI ≥ 25, SBP, LDL-cholesterol, and HbA1c*.

*Adjusted variables for model III: age, sex, BMI≥25, HTN, DM, hypercholesterolemia, and smoking*.

*BMI, body mass index; DM, diabetes mellitus; HbA1C, glycated hemoglobin; HTN, hypertension; LDL, low-density lipoprotein; SBP, systolic blood pressure*.

In model II, the adjusted variables were age groups, gender, BMI≥25, systolic blood pressure, LDL cholesterol, and HbA1C. Both class I and II sarcopenia were still significant parameters in association with increased CAVI (estimate 0.146, SE 0.042, *p* = 0.001 for class I sarcopenia and estimate 0.302, SE 0.073, *p* < 0.010 for class II sarcopenia). Model III included the traditional risk factors of atherosclerosis, which were age groups, gender, BMI ≥ 25, the presence of hypertension, diabetes, hypercholesterolemia, and smoking history. Class I and II sarcopenia were both significant parameters in association with CAVI in model III (estimate 0.148, SE 0.043, *p* = 0.001 for class I sarcopenia and estimate 0.304, SE 0.073, *p* < 0.001 for class II sarcopenia).

## Discussion

In this study, we evaluated the significance of sarcopenia in relation to arterial stiffness in the general Korean population. Our result shows that both class I and II sarcopenia are independently associated with increased arterial stiffness measured by CAVI, even after adjusting for age and traditional cardiovascular risk factors.

Pulse wave velocity has been used as a parameter of arterial stiffness for a long time previously, despite the innate limitation of blood pressure dependence (32–36), as have other parameters of arterial stiffness. Recent data have shown that CAVI has an even stronger association with sarcopenia compared with PWV (7) although the study population has different ethnic backgrounds. The Health, Aging, and Body Composition (Health ABC) study (37) had suggested a racial and gender difference in the link between arterial stiffness and declined muscle mass; Abbatecola et al. found a significant PWV-by-race interaction in women, but not in men. Sex hormones and their association with arterial stiffness is rather complex, especially in the women population with variable menstrual status. Laakkonen et al. reported that age- and hormone-mediated associations in arterial stiffness cannot be differentiated, but still suggested that the potential role of sex hormones as hormonal status was differentially associated with arterial stiffness in age-group focused analyses (38). Overall, there may be a hormonal influence, albeit its power as a cardiovascular risk factor over other traditional risk factors may not be very strong. Although the exact mechanisms to explain the difference among women of different ethnicities were not given, a different hormonal constitution was suggested as one of the possible explanations for gender differences.

Arterial stiffening is caused by complex mechanisms, mainly affected by atherosclerosis. Arterial stiffness and atherosclerosis share some common pathophysiological mechanisms and may be considered synergistic processes that contribute each other in the development of cardiovascular disease (39, 40). Arterial stiffness is an established parameter that reflects the progression of atherosclerosis and, subsequently, cardiovascular disease (41, 42). Most of the erstwhile measurement methods for arterial stiffness had the common limitation that they were affected by blood pressure during measurement, which hindered the clinical application of these methods, especially for a screening purpose. CAVI, a relatively new indicator of arterial stiffness, has overcome such limitation and reflects both functional and organic stiffness; it is relatively independent of blood pressure, with an established role as a screening tool as a measure of arterial stiffness (12, 14, 19, 31, 39, 40, 43).

Some recent studies reported the association between muscle mass and CAVI. CAVI has shown an independent association with skeletal muscle mass by BIA in Japanese men (44). A European Caucasian study from Kirkham et al. (7) also showed that CAVI, among other parameters of vascular stiffness, showed the highest correlation with skeletal mass index, especially in women. Although in a limited population, CAVI has shown a positive association with muscle mass deficit in middle-aged Korean men (21). Im et al.'s study showed that the odds ratio for high CAVI increased with muscle mass deficit in a dose-dependent manner, although the significance disappeared after adjusting for other variables. Our study shows a consistent finding of an independent and significant association between increased CAVI and decreased skeletal muscle mass in the general Korean population. Furthermore, we found that the relationship between sarcopenia and CAVI remained significant after adjusting for traditional cardiovascular risk factors, which can affect arterial stiffness.

The link between muscle mass and arterial stiffness seems to be established although it is still unclear about the conditional relations. Arterial stiffness is considered as an important parameter reflecting endothelial dysfunction, and is also associated with reduced blood flow in the lower extremities in the elderly (45, 46). Some studies suggested that microvascular dysfunction was an explanation to the link, which increased arterial stiffness and subsequent reduced blood flow to the limbs lead to muscle decline (37). From these data, the resulting increased arterial stiffness contributes to higher arterial resistance to blood flow and eventually reduced blood flow to the lower extremities. It may be associated with the development or worsening of decreased muscle mass although the casual relationship of sarcopenia and increased arterial stiffness is still unclear. Some studies have suggested that arterial stiffness was a cause of decreased muscle mass (37), whereas in others, decreased muscle mass was the predisposing factor for increased arterial stiffness (7, 34). In our study, the number of patients with low ABI was not significantly higher in class I or II sarcopenia. At least in our study population, which can be considered as a representative for the general and healthy Korean population, it is more likely that low muscle mass is the predisposing condition related to increased arterial stiffness. Reduced muscle mass is related to low physical activity, insulin resistance and abnormal glucose metabolism, chronic inflammatory state, oxidative stress, and hormonal factors (47–50), which are all significant contributors to increased arterial stiffness.

## Limitations

As our study is cross-sectional, it is still unclear whether this relationship between sarcopenia and arterial stiffness has a causal relationship and which parameter is the cause and which is the consequence. Possible mechanisms have been suggested to explain the link, but the causality or consequences of sarcopenia are yet to be elucidated further. For the arterial stiffness measurement method, we only performed CAVI, and comparison to other measurement methods is not available. However, the advantage of CAVI over other traditional methods is already well known, therefore our center has taken CAVI as a screening tool. We only had BIA data available to determine muscle mass. Other measures evaluating muscle strength or physical performance, which are also important components in the diagnosis of sarcopenia, were not available. Regarding the gender difference on arterial stiffness, our study population is male-predominant and the precise hormonal status of the female population was not available, and thus this parameter could not be evaluated in relation to CAVI. Despite these limitations, our study includes a significant number of the general asymptomatic population with a homogeneous ethnicity with information on comorbidities and medical history to differentiate those with established vascular diseases.

## Conclusion

Low muscle mass is a parameter that is independently and inversely associated with CAVI, adjusting for age and known cardiovascular risk factors. The decline in skeletal muscle mass should be of concern in the assessment of metabolic and cardiovascular risks, even in the apparently healthy population, as CAVI is associated with various metabolic parameters, atherosclerosis, and cardiovascular diseases. Further longitudinal study should be performed to elucidate the causal relationship between sarcopenia and CAVI.

## Data Availability Statement

The original contributions presented in the study are included in the article/supplementary material, further inquiries can be directed to the corresponding author.

## Ethics Statement

The studies involving human participants were reviewed and approved by Institutional Review Board of Seoul National University Hospital (IRB No. H-1606-102-771). Written informed consent for participation was not required for this study in accordance with the national legislation and the institutional requirements.

## Author Contributions

HP and GC made a substantial contribution- to the conception and design of the work, performed the statistical analysis, interpreted the data, and drafted and edited the work as the co-first author. HL contributed to the design and acquisition of the work, interpretation of the data, and drafted the work. MK contributed to the analysis and interpretation of the data, provided comments, and made a revision to the draft. S-YC contributed to the conception and design of the work, analysis, interpretation of the data, and drafted the work. WL made a substantial contribution to the study design, acquisition, statistical analysis, and provided critical comments on the draft. JY as the corresponding author, contributed to the conception, design, acquisition of the work, performed the analysis and interpretation of the data, and drafted the work. All authors contributed to the article and approved the submitted version.

## Conflict of Interest

The authors declare that the research was conducted in the absence of any commercial or financial relationships that could be construed as a potential conflict of interest.

## Publisher's Note

All claims expressed in this article are solely those of the authors and do not necessarily represent those of their affiliated organizations, or those of the publisher, the editors and the reviewers. Any product that may be evaluated in this article, or claim that may be made by its manufacturer, is not guaranteed or endorsed by the publisher.

## References

[1] Dutta C. Significance of sarcopenia in the elderly. J Nutr. (1997) 127:992S−3S. 10.1093/jn/127.5.992S 9164281

[2] Visser M Kritchevsky SB Goodpaster BH Newman AB Nevitt M Stamm E. Leg muscle mass and composition in relation to lower extremity performance in men and women aged 70 to 79: the health, aging and body composition study. J Am Geriatr Soc. (2002) 50:897–904. 10.1046/j.1532-5415.2002.50217.x 12028178

[3] Sasaki H Kasagi F Yamada M Fujita S. Grip strength predicts cause-specific mortality in middle-aged and elderly persons. Am J Med. (2007) 120:337–42. 10.1016/j.amjmed.2006.04.018 17398228

[4] Kinney JM. Nutritional frailty, sarcopenia and falls in the elderly. Curr Opin Clin Nutr Metab Care. (2004) 7:15–20. 10.1097/00075197-200401000-00004 15090898

[5] Stephens JW Khanolkar MP Bain SC. The biological relevance and measurement of plasma markers of oxidative stress in diabetes and cardiovascular disease. Atherosclerosis. (2009) 202:321–9. 10.1016/j.atherosclerosis.2008.06.006 18640680

[6] Brillante DG O'Sullivan AJ Howes LG. Arterial stiffness in insulin resistance: the role of nitric oxide and angiotensin II receptors. Vasc Health Risk Manag. (2009) 5:73–8. 10.2147/VHRM.S3784 19436651 PMC2672440

[7] Kirkham FA Bunting E Fantin F Zamboni M. Rajkumar C. Independent association between cardio-ankle vascular index and sarcopenia in older UK adults. J Am Geriatr Soc. (2019) 67:317–22. 10.1111/jgs.15648 30460978

[8] Hougaku H Fleg JL Najjar SS Lakatta EG Harman SM Blackman MR. Relationship between androgenic hormones and arterial stiffness, based on longitudinal hormone measurements. Am J Physiol Endocrinol Metab. (2006) 290:E234–42. 10.1152/ajpendo.00059.2005 16159908

[9] Campos AM Moura FA Santos SN Freitas WM Sposito AC Brasilia Brasilia Study on Healthy A Brasilia Heart S. Sarcopenia, but not excess weight or increased caloric intake, is associated with coronary subclinical atherosclerosis in the very elderly. Atherosclerosis. (2017) 258:138–44. 10.1016/j.atherosclerosis.2017.01.005 28129889

[10] Van Bortel LM Laurent S Boutouyrie P Chowienczyk P Cruickshank JK Backer TD. Expert consensus document on the measurement of aortic stiffness in daily practice using carotid-femoral pulse wave velocity. J Hypertens. (2012) 30:445–8. 10.1097/HJH.0b013e32834fa8b0 22278144

[11] Benetos A Okuda K Lajemi M Kimura M Thomas F Skurnick J. Telomere length as an indicator of biological aging: the gender effect and relation with pulse pressure and pulse wave velocity. Hypertension. (2001) 37:381–5. 10.1161/01.HYP.37.2.381 11230304

[12] Shirai K Utino J Otsuka K Takata M. A novel blood pressure-independent arterial wall stiffness parameter; cardio-ankle vascular index (CAVI). J Atheroscler Thromb. (2006) 13:101–7. 10.5551/jat.13.101 16733298

[13] Choi SY Oh BH Park JB Choi DJ Rhee MY Park S. Age-associated increase in arterial stiffness measured according to the cardio-ankle vascular index without blood pressure changes in healthy adults. J Atheroscler Thromb. (2013) 20:911–23. 10.5551/jat.18267 23965527

[14] Park HE Choi SY Kim MK Oh BH. Cardio-ankle vascular index reflects coronary atherosclerosis in patients with abnormal glucose metabolism: assessment with 256 slice multi-detector computed tomography. J Cardiol. (2012) 60:372–6. 10.1016/j.jjcc.2012.07.005 22890071

[15] Kim H Kim HS Yoon HJ Park HS Cho YK Nam CW. Association of cardio-ankle vascular index with diastolic heart function in hypertensive patients. Clin Exp Hypertens. (2014) 36:200–5. 10.3109/10641963.2013.804544 23786431

[16] Chung GE Park HE Lee H Choi SY. Clinical significance of increased arterial stiffness associated with atrial fibrillation, according to Framingham risk score. Sci Rep. (2021) 11:4955. 10.1038/s41598-021-84311-9 33654162 PMC7925576

[17] Choi SY Park HE Seo H Kim M Cho SH Oh BH. Arterial stiffness using cardio-ankle vascular index reflects cerebral small vessel disease in healthy young and middle aged subjects. J Atheroscler Thromb. (2013) 20:178–85. 10.5551/jat.14753 23131963

[18] Park HE Choi SY Kim HS Kim MK Cho SH Oh BH. Epicardial fat reflects arterial stiffness: assessment using 256-slice multidetector coronary computed tomography and cardio-ankle vascular index. J Atheroscler Thromb. (2012) 19:570–6. 10.5551/jat.12484 22472214

[19] Park HE Lee H Choi SY Kwak MS Yang JI Yim JY. Usefulness of controlled attenuation parameter for detecting increased arterial stiffness in general population. Dig Liver Dis. (2018) 50:1062–7. 10.1016/j.dld.2018.04.027 29779697

[20] Tap L Kirkham FA Mattace-Raso F Joly L Rajkumar C Benetos A. Unraveling the links underlying arterial stiffness, bone demineralization, and muscle loss. Hypertension. (2020) 76:629–39. 10.1161/HYPERTENSIONAHA.120.15184 32755468

[21] Im IJ Choi HJ Jeong SM Kim HJ Son JS Oh HJ. The association between muscle mass deficits and arterial stiffness in middle-aged men. Nutr Metab Cardiovasc Dis. (2017) 27:1130–5. 10.1016/j.numecd.2017.10.002 29170061

[22] Silva AM Shen W Heo M Gallagher D Wang Z Sardinha LB. Ethnicity-related skeletal muscle differences across the lifespan. Am J Hum Biol. (2010) 22:76–82. 10.1002/ajhb.20956 19533617 PMC2795070

[23] Larson S Cho MC Tsioufis K Yang E. 2018 Korean Society of Hypertension Guideline for the Management of Hypertension: A Comparison of American, European, and Korean Blood Pressure Guidelines. Eur Heart J. (2020) 41:1384–6. 10.1093/eurheartj/ehaa114 32259269

[24] American Diabetes Association. 2. Classification and Diagnosis of Diabetes: Standards of Medical Care in Diabetes-−2021. Diabetes Care. (2021) 44:S15–33. 10.2337/dc21-S002 33298413

[25] Cho SMJ Lee H Lee HH Baek J Heo JE Joo HJ. on behalf of the Korean Society of Lipid and Atherosclerosis (KSoLA) Public Relations Committee. Dyslipidemia Fact Sheets in Korea 2020: an analysis of nationwide population-based data. J Lipid Atheroscler. (2021) 10:202–9. 10.12997/jla.2021.10.2.202 34095012 PMC8159761

[26] Rhee EJ Kim HC Kim JH Lee EY Kim BJ Kim EM. 2018 Guidelines for the management of dyslipidemia. Korean J Intern Med. (2019) 34:723–71. 10.3904/kjim.2019.188 31272142 PMC6610190

[27] Chung GE Park HE Lee H Kim MJ Choi SY Yim JY. Sarcopenic obesity is significantly associated with coronary artery calcification. Front Med. (2021) 8:651961. 10.3389/fmed.2021.651961 33855037 PMC8039284

[28] Chung GE Kim MJ Yim JY Kim JS Yoon JW. Sarcopenia is significantly associated with presence and severity of nonalcoholic fatty liver disease. J Obes Metab Syndr. (2019) 28:129–38. 10.7570/jomes.2019.28.2.129 31294345 PMC6604841

[29] Janssen I Heymsfield SB Ross R. Low relative skeletal muscle mass (sarcopenia) in older persons is associated with functional impairment and physical disability. J Am Geriatr Soc. (2002) 50:889–96. 10.1046/j.1532-5415.2002.50216.x 12028177

[30] Kim YS Lee Y Chung YS Lee DJ Joo NS Hong D. Prevalence of sarcopenia and sarcopenic obesity in the Korean population based on the Fourth Korean National Health and Nutritional Examination Surveys. J Gerontol A Biol Sci Med Sci. (2012) 67:1107–13. 10.1093/gerona/gls071 22431554

[31] Park JB Park HE Choi SY Kim MK Oh BH. Relation between cardio-ankle vascular index and coronary artery calcification or stenosis in asymptomatic subjects. J Atheroscler Thromb. (2013) 20:557–67. 10.5551/jat.15149 23524474

[32] Yamanashi H Kulkarni B Edwards T Kinra S Koyamatsu J Nagayoshi M. Association between atherosclerosis and handgrip strength in non-hypertensive populations in India and Japan. Geriatr Gerontol Int. (2018) 18:1071–8. 10.1111/ggi.13312 29582539 PMC6144064

[33] Kohara K Okada Y Ochi M Ohara M Nagai T Tabara Y. Muscle mass decline, arterial stiffness, white matter hyperintensity, and cognitive impairment: Japan Shimanami Health Promoting Program study. J Cachexia Sarcopenia Muscle. (2017) 8:557–66. 10.1002/jcsm.12195 28371474 PMC5566649

[34] Ohara M Kohara K Tabara Y Ochi M Nagai T Igase M. Sarcopenic obesity and arterial stiffness, pressure wave reflection and central pulse pressure: the J-SHIPP study. Int J Cardiol. (2014) 174:214–7. 10.1016/j.ijcard.2014.03.194 24767131

[35] Kim TN Park MS Lim KI Yang SJ Yoo HJ Kang HJ. Skeletal muscle mass to visceral fat area ratio is associated with metabolic syndrome and arterial stiffness: The Korean Sarcopenic Obesity Study (KSOS). Diabetes Res Clin Pract. (2011) 93:285–91. 10.1016/j.diabres.2011.06.013 21752483

[36] ZhangLGuoQFengBLWangCYHanPPHuJ. A cross-sectional study of the association between arterial stiffness and sarcopenia in chinese community-dwelling elderly using the asian working group for sarcopenia criteria. J Nutr Health Aging. (2019) 23:195–201. 10.1007/s12603-018-1147-930697630

[37] Abbatecola AM Chiodini P Gallo C Lakatta E Sutton-Tyrrell K Tylavsky FA. Pulse wave velocity is associated with muscle mass decline: health ABC study. Age (Dordr). (2012) 34:469–78. 10.1007/s11357-011-9238-0 21479573 PMC3312626

[38] Laakkonen EK Karppinen JE Lehti S Lee E Personen E Juppi HK. Associations of sex hormones and hormonal status with arterial stiffness in a female sample from reproductive years to menopause. Front Endocrinol. (2021) 12:765916 10.3389/fendo.2021.765916 34917027 PMC8669797

[39] Sun CK. Cardio-ankle vascular index (CAVI) as an indicator of arterial stiffness. Integr Blood Press Control. (2013) 6:27–38. 10.2147/IBPC.S34423 23667317 PMC3650513

[40] Shirai K. Analysis of vascular function using the cardio-ankle vascular index (CAVI). Hypertens Res. (2011) 34:684–85. 10.1038/hr.2011.40 21642994

[41] Oliver JJ Webb DJ. Noninvasive assessment of arterial stiffness and risk of atherosclerotic events. Arterioscler Thromb Vasc Biol. (2003) 23:554–66. 10.1161/01.ATV.0000060460.52916.D6 12615661

[42] O'Rourke MF Staessen JA Vlachopoulos C Duprez D Plante GE. Clinical applications of arterial stiffness; definitions and reference values. Am J Hypertens. (2002) 15:426–44. 10.1016/S0895-7061(01)02319-6 12022246

[43] Kubozono T Miyata M Ueyama K Nagaki A Otsuji Y Kusano K. Clinical significance and reproducibility of new arterial distensibility index. Circ J. (2007) 71:89–94. 10.1253/circj.71.89 17186984

[44] Sampaio RA Sampaio PY Yamada M Yukutake T Uchida MC Tsuboyama T. Arterial stiffness is associated with low skeletal muscle mass in Japanese community-dwelling older adults. Geriatr Gerontol Int. (2014) 14:109–14. 10.1111/ggi.12206 24450568

[45] Suzuki E Kashiwagi A Nishio Y Egawa K Shimizu S Maegawa H. Increased arterial wall stiffness limits flow volume in the lower extremities in type 2 diabetic patients. Diabetes Care. (2001) 24:2107–14. 10.2337/diacare.24.12.2107 11723092

[46] Mitchell GF Lacourciere Y Arnold JM Dunlap ME Conlin PR Izzo JL Jr. Changes in aortic stiffness and augmentation index after acute converting enzyme or vasopeptidase inhibition. Hypertension. (2005) 46:1111–7. 10.1161/01.HYP.0000186331.47557.ae 16230523

[47] Ochi M Kohara K Tabara Y Kido T Uetani E Ochi N. Arterial stiffness is associated with low thigh muscle mass in middle-aged to elderly men. Atherosclerosis. (2010) 212:327–32. 10.1016/j.atherosclerosis.2010.05.026 20554280

[48] Cesari M Kritchevsky SB Baumgartner RN Atkinson HH Penninx BW Lenchik L. Sarcopenia, obesity, and inflammation–results from the Trial of Angiotensin Converting Enzyme Inhibition and Novel Cardiovascular Risk Factors study. Am J Clin Nutr. (2005) 82:428–34. 10.1093/ajcn/82.2.428 16087989

[49] Cruz-Jentoft AJ Baeyens JP Bauer JM Boirie Y Cederholm T Landi F. Sarcopenia: European consensus on definition and diagnosis: report of the European Working Group on Sarcopenia in Older People. Age Ageing. (2010) 39:412–23. 10.1093/ageing/afq034 20392703 PMC2886201

[50] Srikanthan P Hevener AL Karlamangla AS. Sarcopenia exacerbates obesity-associated insulin resistance and dysglycemia: findings from the National Health and Nutrition Examination Survey III. PLoS ONE. (2010) 5:e10805. 10.1371/journal.pone.0010805 22421977 PMC3279294

